# Family Structure, Unstructured Socializing, and Heavy Substance Use among Adolescents

**DOI:** 10.3390/ijerph19148818

**Published:** 2022-07-20

**Authors:** John P. Hoffmann

**Affiliations:** Department of Sociology, Brigham Young University, Provo, UT 84602, USA; john_hoffmann@byu.edu

**Keywords:** family structure, single parent, unstructured socializing, heavy alcohol use, heavy substance use

## Abstract

Background: Psychoactive substance use is a transient behavior among many adolescents and diminishes as they mature, but some engage in heavy forms of substance use, which increases their risk of health and behavioral challenges. A consistent predictor of substance use among youth is family structure, with adolescents living in single-parent, stepparent, or no-parent families at higher risk than others of several forms of substance use. The objective of this research was to investigate whether unstructured socializing mediated the association between family structure and heavy alcohol or substance use. Methods: Data from 30 nations (*n* = 65,737) were used to test the hypothesis using a generalized structural equation model and tests of mediation. Results: The analysis furnished clear support for a mediation effect among adolescents living with a single parent but less support among those living with a stepparent or neither parent. Conclusion: The association between living in a single-parent household and heavy alcohol or other substance use was mediated largely by time spent outside the home with friends in unsupervised activities. Additional research that uses longitudinal data and more nuanced measures of family structure is needed to validate this finding.

## 1. Introduction

Psychoactive substance use among adolescents is an international health concern [1,2]. For most youth, experimenting with psychoactive substances, such as using cannabis, stimulants, or hallucinogens, is a transient behavior and diminishes as they mature. However, some youth engage in heavy forms of substance use, and this increases their risk of substance use disorders (SUDs) as well as life course impediments, such as academic problems, impaired cognitive functioning and brain development, and mental health challenges [3,4,5,6,7,8].

Recent international data have shown that cannabis use increased across the globe during the last two decades, which is not surprising given that many nations have decriminalized or legalized its consumption. However, this upward trend has occurred as more potent forms of cannabis have become available. It has also been accompanied by a higher prevalence among adolescents even in those nations in which use is sanctioned only for adults [2]. Yet, most experts and policy bodies recommend that youth avoid cannabis because of its detrimental effects on development [9,10]. Use of other euphoriant substances also increased among adolescents and young adults in recent years, with a growing number in treatment for an opioid use disorder and a 35 percent increase in deaths due to substance use disorders [2,11]. Alcohol use among adolescents, on the other hand, has decreased in most nations though the prevalence of heavy use remains above 20% in several European and North American countries [12,13]. Clearly, research on adolescent substance use, especially heavier forms, continues to be relevant.

Studies of substance use and consequent problems among young people have focused largely on peers, sociocultural conditions, and neurological factors [14,15]. Research continues to show, however, that family characteristics affect the course of substance use [16,17,18]. In addition to family functioning, many studies have examined family structure’s association with substance use among youth. Research conducted over the last 25 years has shown consistently that adolescents living with a single mother, a single father, or neither parent are at higher risk than those living with two parents of general substance use as well as problem use [19,20,21,22,23,24,25,26,27]. Studies have also found that living with a stepparent is associated with more frequent substance use among young people [21,22,23,24].

Whether two-parent families offer some protection against substance use takes on greater prominence when recognizing that the percentage of adolescents living with both biological parents has decreased in most developed countries [28,29]. The growth of single-parent and stepparent families in many nations may thus herald increasing substance use among youth in some parts of the world. On the other hand, perhaps as two-parent families lose their normative status, and the stigmatization of alternative living arrangements fades, the effects of family structure on youth substance use will diminish.

Few scholars believe that family structure has a direct or uniform influence on adolescent substance use. Instead, most studies have attempted to determine the mechanisms through which living with, for example, a single father or single mother increases the risk of substance use. Much of this research has addressed socioeconomic conditions such as family income or living in poverty; family relationships such as parent–child warmth, communication, and parenting styles that affect the socialization of youth; and the stress that often accompanies family dissolution or reconfiguration [22,24,30,31,32,33]. For example, many single-mother families have relatively few economic resources, which affects the parent’s ability to invest in their children [30,33]. Stepparents, though they tend to experience less economic hardships than single parents [34], are not as apt to develop close emotional ties or supervise their stepchildren [35,36]. Studies of kinship suggest that biological affinity is associated with many positive developmental outcomes among youth [37] but is not fully available in stepparent families. Finally, scholars have posited that the stress produced by family reconfiguration may lead some youth to substance use as a way of coping with the pressures they feel at home. The euphoria produced by psychoactive substances can furnish a way for young people to alleviate feelings of distress, anxiety, and negative affect due to adverse family experiences and other untoward events [38,39,40].

### 1.1. Unstructured Socializing and Adolescent Substance Use

A recent addition to the list of factors that could account for the family structure–adolescent substance use association involves unstructured socializing. Research has established that most youth who engage in substance use do so with friends and that peer use is a key proximal predictor of one’s own use [41], but the context within which peer relations take place is important. Rooted in routine activities theory [42], unstructured socializing refers to time spent with friends outside the presence of adults but with no specific plans or structure [43]. A key notion underlying this concept is that as youth spend more unplanned time with friends but without supervision, their opportunities to mis-behave increase. Assuming these opportunities exist, misbehaviors tend to be more rewarding because they demonstrate group cohesion and commitment to one’s peers. Because responsible parents or adult authority figures are not present, we may also expect that monitoring of behaviors—a key aspect of social control theories of deviance—is absent, and the likelihood of misbehaviors increases. Finally, as individuals experience more unstructured time outside the home, they are increasingly likely to associate with deviant peers, which also increases the risk of misbehaviors [44,45].

Although unstructured socializing is related to a lack of parental supervision or monitoring, and thus, some may see these two concepts as polar yet redundant, they differ because unstructured activities tend to be under the volition of the youth. Whereas parents must make an effort to supervise their children’s activities, young people usually use their agency to select activities where adults are not present or that are not organized. Of course, parents can set limits on unstructured socializing by managing their children’s activities, but some youth can circumvent these efforts if they choose to.

Consistent with this perspective, research has suggested that adolescents who spend relatively more time with friends—and less time at home—in unsupervised activities are at higher risk of various types of substance use [44,46,47,48]. Although not yet established, it seems likely that because, as noted earlier, time spent with and supervision of youth are attenuated in single-parent and stepparent families [33,35,49], the likelihood of unstructured socializing increases. This suggests that unstructured socializing may mediate part of the association between family structure and adolescent substance use.

Studies have not yet explored whether this proposition is valid. However, Hoffmann [22], using data from 37 nations to identify relationships between family structure and frequency of alcohol and cannabis use, determined that time spent with friends attenuated the association between living in a single- or stepparent family and alcohol use by about 30 percent. The empirical model did not clarify, however, whether this attenuation was due to confounding or a mediation effect. Hoffmann tested only whether parent–child relationships mediated the association between family structure and youth substance use (they did not). An interesting aspect of this study was that the empirical models also considered whether socioeconomic resources, parent–child relationships, or stress affected the family structure–substance use association, but they had no discernible effects cf. [30,50].

### 1.2. The Current Study

Considering Hoffmann’s [22] intriguing yet conceptually and empirically underdeveloped attention to unstructured socializing, the goal of this research was to assess whether the family structure–adolescent substance use association is mediated in part by time spent by youth with peers in unsupervised activities. Since, as noted earlier, substance use for many youth is a transitory behavior that poses few long-term risks, the outcomes of interest are heavy alcohol and other substance use behaviors that increase the risk of negative developmental consequences [7,51]. Finally, in accord with recent efforts to explore the association between family structure and youth behaviors cross-nationally [19,22], this research utilized data from 30 countries that participated in the Second International Self-Report Delinquency Study (ISRD-2) [52,53]. Using data from several nations alleviates some of the concerns that social science research findings have a Western or U.S. bias.

The hypothesis guiding this research was therefore:

**Hypothesis** **1** **(H_1_).** *The association between family structure and heavy substance use among adolescents is mediated by unstructured socializing.*

The hypothesis did not specify whether specific family structures—such as single-parent or stepparent families—are more or less likely to be affected by unstructured socializing. One might surmise, however, that youth from single-parent families are most likely to engage in unstructured socializing and consequently be at risk of heavy forms of use.

## 2. Material and Methods

### 2.1. Procedures and Participants

The data used to assess the hypothesis were from the ISRD-2, a cross-national study of delinquency and substance use among from 7th to 12th grade students in 30 nations. The nations included 25 European countries, the United States, Suriname, Venezuela, and two Caribbean islands affiliated with the Netherlands: Netherlands Antilles and Aruba. Students were selected by probability samples of classrooms from schools that were in cities and towns considered “typical” by the research team or in large cities. Standard sampling protocols and questionnaires were administered in each nation to ensure that respondents answered the same survey questions. The surveys were collected between 2005 and 2007, attaining an overall sample size (ignoring missing data) of 73,396. The researchers estimated that the overall response rate was about 74 percent [52,53]. The questionnaires and codebooks from the study are available at https://www.icpsr.umich.edu/web/ NACJD/studies/34658/datadocumentation (accessed on 20 June 2022).

### 2.2. Measures

The outcome variables, namely heavy alcohol use and heavy substance use, were operationalized by a series of questions that asked adolescent respondents about their lifetime and past month use of alcohol (beer, wine, or liquor/spirits), cannabis, ecstasy, speed, LDS, heroin, cocaine, or similar psychoactive substances. Frequency questions regarding the last month included the number of drinks or times used. The alcohol questions were utilized to create a variable that included the following categories: never used, used but more than a month ago, used in the last month but no heavy use, and heavy use in the last month. Using a categorical variable that incorporates different levels of alcohol use and includes options that exhaust all possibilities of use obviates the likelihood of selection effects in empirical models [54]. Heavy alcohol use was defined as drinking four or more drinks (females) or five or more drinks (males) the last time one drank in the past month, a measurement strategy similar to other studies using the ISRD-2 data [55] but different than other measures of heavy use that usually specify the number of drinks per occasion during a two-week period [56]. Comparing the percent of heavy alcohol users among 12–17-year-olds in the U.S. from the 2005 National Survey on Drug Use and Health (NSDUH) to those in the ISRD-2 sample from the U.S. revealed close estimates, however. Across the ISRD-2 nations, almost 10% of youth reported heavy alcohol use.

Heavy substance use was measured in a similar manner as heavy alcohol use, but the frequency of use specified five or more times using cannabis and/or four or more times using another illicit substance such as cocaine, ecstasy, or stimulants in the previous month. About 90% of youth reported they had never used any of these substances, and about 2.5% reported heavy use in the past month. Table 1 provides the percentages for each category of alcohol and illicit substance use. Appendix A lists the percent of youth who reported heavy alcohol and substance use by country.

Family structure was identified using a single questionnaire item that asked youth whether they were living with their own mother and father. The responses included “yes, I live with my own mother and father” as well as eight other options (e.g., live part time with each parent; live with only one’s mother; live with one’s mother and a stepfather). The variable was coded so respondents were placed into one of eight categories: mother-father, joint custody, mother only, father only, mother–stepfather, father–stepmother, neither parent, and other family type. Note that multigenerational families, adoption, or same-gender parenting arrangements could not be uniquely identified in the ISRD-2 data. In the empirical models, mother–father families served as the reference category.

The hypothesis proposed that unstructured socializing mediates the association between family structure and heavy alcohol/substance use. Matching previous studies using the ISRD-2 data [57], this variable was measured with a set of items that asked youth how often they go out at night to parties, dance clubs, or hang out on the street; how much time they hang out with friends; how often they spend free time with friends; how frequently they and their friends spend in public places; and how often they and their friends hang out at dance clubs or music concerts. Each variable was coded using categorical response options—including yes/no and ordered responses (e.g., never, sometimes, often, always)—so higher values indicated more time “hanging out”. The five variables were subjected to a principal components analysis (PCA) designed for categorical indicators [58]. Based on the Guttman–Kaiser criterion, a single latent variable emerged from the PCA, which accounted for about 78% of the shared variability among the items. The omega reliability coefficient [59] for the scale was 0.68. This measurement scheme assumes that much of their time “hanging out” is unsupervised though there are probably exceptions [57].

Other variables included in the model were drawn from previous research on family structure and adolescent behaviors, youth substance use, and the correlates of delinquency and substance use using the ISRD-2 data. The first set was based on studies suggesting that family structure might be linked to adolescent problem behaviors due to the former’s connections to attenuated economic resources, parent–child relationship, or parental supervision as well as life stressors [19,22,23,24,31,38,40]. Substance use research has also highlighted the role of low self-control and peer substance use as predictors of heavy alcohol and substance use [46,48] though low self-control might instead reflect risk-taking as an adaptive strategy. In addition to these factors, studies using the ISRD-2 data have noted that neighborhood characteristics and immigration status are associated with adolescent alcohol use [53]. Immigration status is a common stressor among adolescents and thus might affect the risk of substance use.

Economic resources were based on four items that asked youth whether they had a room of their own, a computer at home, owned a mobile phone, or if their family owned a car (“yes” coded as zero, and “no” coded as one). The sum of the items was used to gauge low economic resources. The KR20 reliability coefficient of the items was 0.59 [60]. The ISRD-2 data did not include traditional measures of economic resources or socioeconomic status such as income or wealth.

To instantiate parent–child relationships, a set of questionnaire items was used that assessed how well respondents got along with their mother, stepmother, father, or stepfather (whichever was applicable). The response options included “not at all”, “not so well”, “rather well”, and “very well.” A PCA designed for categorical variables revealed that a single latent construct accounted for 79% of the shared variance among the items. The omega reliability coefficient for the scale was 0.67.

Family involvement was gauged by two items that inquired about how often respondents did things with their parents or adults in the home, such as going to the movies, sporting events, on a hike, or similar activities, and how many days in a typical week they had an evening meal with parents and adults in the home. The first variable’s response options ranged from “almost never” to “more than once a week”, whereas the second’s ranged from “never” to “daily.” The latent construct from a PCA accounted for 65% of the shared variance. The omega reliability coefficient was 0.59.

Parental supervision was based on questions that asked whether their parents knew who they were with when they went out and told them what time to be home in the evening. Response options for the first item included “rarely/never”, “sometimes”, and “always”. For the second item, the response options included “no”, “rarely”, “sometimes”, and “always”. A PCA revealed a single latent construct accounting for 62% of the shared variance. The omega reliability coefficient was 0.57.

Stressful life events were quantified as a count variable based on nine questions that inquired about whether youth had experienced the death of or a long or serious illness among a sibling, parent, or someone they loved; physical conflicts among adults in the home; a separation or divorce; parents having problems finding a job; or discrimination because of their color, religion, or language spoken. The range of the variable was thus 0–9, with a little more than half the respondents experiencing zero or only one event. Given the outcome variables, a separate binary variable was included based on a question about whether a parent or stepparent had problems with alcohol or drugs [61]. Almost eight percent of respondents reported parent or stepparent drug problems.

The ISRD-2 data included twelve questions from Grasmick and colleagues’ self-control scale, which gauged impulsivity, risk-taking, temper, and self-centeredness (e.g., “I act on the spur of the moment without stopping to think”; “I’m more concerned with what happens to me in the short run than in the long run”) [62]. The four response options ranged from “fully disagree” to “fully agree”. The items were subjected to a PCA for categorical variables, which revealed that a single latent construct accounted for 88% of the shared variance. Higher values indicated lower self-control. The omega reliability coefficient was 0.83.

Peer substance use was based on a single question asking respondents to report the number of their friends who used substances such as “weed, hash, XTC, speed, heroin, or coke”. (The question did not include alcohol or tobacco.) The variable was highly skewed (skewness = 2.3), so its natural logarithm was computed and used as the measure of peer substance use (skewness = 1.5).

The second set of control variables consisted of biological sex (female vs. male), age in years, whether the respondent was an immigrant, whether their parents were immigrants, and a measure of neighborhood quality constructed for the ISRD-2 public use file. It was based on a set of 13 questions that asked whether respondents liked their neighborhood; thought it was a close knit, helpful, and trusting community; and their perceptions of neighborhood crime, drug selling, and graffiti [53]. The variable’s scale ranged from 0–100, with higher values indicating a better-quality neighborhood.

Finally, in line with cross-national studies of youth substance use and family structure [19,22], the empirical models included the following variables measured at the nation-level: estimates of the percent of youth living in single parent families [28], liters of alcohol used per capita [63], and the percent of cannabis users among the adult population [64].

Various analyses could not determine whether the data used in the analyses were missing at random. Therefore, complete case analysis was conducted rather than estimating models with multiply imputed datasets [65]. Note, though, that the overall missingness percentage was less than 10%. Table 1 furnishes summary statistics for all the variables used in the analyses.

### 2.3. Empirical Model

Because the data consisted of individuals nested in 30 nations, and the hypothesis focused on the mediating effect of unstructured socializing, multilevel structural equation models (SEMs) were utilized [66]. The two outcomes—heavy alcohol use and heavy substance use—were multinomial variables, so the analyses relied on generalized SEMs designed for categorical and continuous variables. All analyses considered the complex sampling design of the ISRD-2 and were based on weighted data. Because measures of model fit for generalized SEMs are not well-established, the goodness-of-fit statistic utilized for the models was the *true-positive rate* (TPR). Also called *model sensitivity*, the TPR assesses the probability of a positive test, in this case a respondent predicted to be a heavy user, conditioned on truly being positive or reporting actual heavy use.

$$TPR=\frac{\mathrm{Number}\;\mathrm{of}\;\mathrm{accurate}\;\mathrm{predictions}\;\mathrm{from}\;\mathrm{model}}{\mathrm{Total}\;\mathrm{number}\;\mathrm{of}\;\mathrm{heavy}\;\mathrm{users}}$$

The first set of SEMs estimated the direct effects of family structure on heavy alcohol use and heavy substance use with multinomial logistic regression models. Only the results for the heavy use vs. no use categories are included in the tables although all the outcome categories were included in the models. The second set of SEMs examined the direct effects of family structure on heavy use along with unstructured socializing and all the control variables. These results are presented in Table 2 and Table 3.

The third set of SEMs examined two simultaneous models: the estimated effects of family structure and the control variables on unstructured socializing using linear models and the association between all variables and heavy use using multinomial logistic models (see Table 4 and Table 5). They were designed to explicitly show the direct effects on the two variables. The predictors of unstructured socializing included parental supervision. This was justified by research suggesting that when parents do not monitor their children’s whereabouts, the likelihood of unstructured socializing increases [67].

Mediation models are typically interested in decomposing the effects of a predictor on an outcome into full, direct, and indirect effects. This issue is complicated, however, when the empirical models are of different types, such as a linear and a multinomial model [68,69]. Two approaches were utilized to address this complication. First, a bootstrap approach was employed to compute direct and indirect effects and their confidence intervals (CIs) following model estimation (see Table 6).

Second, as a validation exercise for shifts in family structure effects across the multinomial models and the mediation models, the KHB method for decomposing regression coefficients was used. This method residualizes the mediator of a suspected confounder with respect to the predictors and uses a standardization approach to make the coefficients comparable. The method is thus suitable for estimating total, direct, and indirect effects as well as confounding across nested regression models [70]. However, the KHB procedure relies on the delta method to estimate standard errors and CIs, which simulation studies have shown is suboptimal relative to the bootstrap [71,72]. Convergence of results, nonetheless, should demonstrate that the direct and indirect effects of family structure on heavy alcohol use are properly specified.

## 3. Results

### 3.1. Heavy Alcohol Use

Table 2 presents the results of the initial SEMs designed to estimate the association between family structure and heavy alcohol use. As mentioned earlier, the table shows the results only for heavy alcohol use versus the comparison group of no lifetime alcohol use. The family structure coefficients, presented as odds ratios (ORs), compare the group listed to the reference group of mother–father families.

Model 1 examined the direct association between family structure and heavy alcohol use. Youth from each type of family manifested a higher odds of heavy alcohol use relative to those living with their mother and father. For example, the odds of heavy alcohol use among youth in single-father families were estimated at about 83% higher than among those in mother–father families, whereas the odds for youth in mother–stepfather or neither-parent families were estimated to be almost two-and-a-half times those for youth in mother–father families.

Model 2 added the control variables and unstructured socializing. Consistent with the hypothesis, though not providing direct evidence of its validity, the family structure effects were attenuated substantially. For example, the odds ratio gauging the association between single-father families and heavy alcohol use dropped from 1.83 to 0.92, and the latter’s 95% CI included one (0.67, 1.27). The odds ratios associated with joint custody and mother–stepfather families remained substantial, however (OR, joint custody: 1.45 (95% CI: 1.04, 2.02); OR, mother–stepfather: 1.61 (95% CI: 1.31, 1.98)), though they also diminished from model 1 to model 2. Since nested multinomial logistic regression coefficients are challenging to compare across models due to dissimilar variances, the reduced associations were confirmed using the KHB method [70]. According to the guiding hypothesis, the reduction in these family structure effects was likely due to the mediating effect of unstructured socializing.

Model 2 also demonstrated an association between parent drug problems, low self-control, peer substance use, or unstructured socializing and an elevated risk of heavy alcohol use. The association with peer substance use was, not surprisingly, especially large (OR = 4.51) though the variable’s scale was compressed because it was measured in log-units, so comparing its effect size to others is not prudent.

As expected, unstructured socializing had a notable association with heavy alcohol use (OR = 2.06; 95% CI: 1.84, 2.32). Youth who spent more time hanging out with friends in public places or at dance venues and concerts were more likely to report heavy alcohol use even with statistical adjustments for parental supervision, parents’ drug problems, low self-control, and peer substance use. The TPV improved substantially when all the variables were included, rising from 0.33 to 0.78. In other words, model 2 correctly predicted about 78% of heavy alcohol users.

The results shown in Table 3 extend those in Table 2 by furnishing the direct effects of family structure and a selection of the control variables on unstructured socializing. The coefficients in the first unstructured socializing column are beta weights, with *y*-standardization used for categorical predictors and full standardization used for continuous predictors. For example, compared with youth who lived with their mother and father, those residing in single-mother families were predicted to be about 0.11 standard deviations higher on the unstructured socializing scale. Four family structure categories were related to unstructured socializing: joint custody, single-mother, single-father, and mother–stepfather; youth in these family types were more likely to engage in unstructured socializing relative to youth who lived in mother–father families. According to the heavy alcohol use columns, once unstructured socializing and other factors were evaluated, only youth from joint custody and mother–stepfather families manifested an elevated risk of heavy alcohol use.

### 3.2. Heavy Substance Use

Table 4 and Table 5 provide parallel analyses of family structure, unstructured socializing, and heavy substance use—cannabis, ecstasy, cocaine, speed, and others. With a few exceptions, the results matched those from the heavy alcohol use models. Youth from joint custody, single-mother, single-father, mother–stepfather, and neither-parent families manifested a higher risk of heavy substance use than those from mother–father families (see Table 4). However, once unstructured socializing and the control variables were introduced, the associations with heavy substance use diminished considerably, and the 95% CIs for three of the five family structures’ ORs included one. Once again, the KHB method [70] confirmed these reductions. Of note, however, is the relatively large odds ratio for youth who lived with neither parent (OR = 2.45): the odds of heavy substance use were more than double those of youth who lived with their mother and father even after adjusting for the effects of unstructured socializing, peer substance use, and other factors.

Table 5 shows the associations of family structure with unstructured socializing. Again, the results are consistent with the earlier model. Youth living in joint custody, single-mother, single-father, and mother–stepfather families tended to report more time hanging out with friends at clubs, parties, and in public places. Moreover, parental supervision was negatively associated with unstructured socializing (β = −0.29; 95% CI: −0.32, −0.27): youth who reported their parents knew with whom they were out and set a time to come home were apt to spend less time in unstructured socializing.

### 3.3. Direct and Indirect Effects of Family Structure

Table 6 furnishes the direct effects of family structure on unstructured socializing and heavy use and the indirect effects of family structure on heavy use. The utility of these results is in evaluating what portion of the effects of the family structure variables on heavy alcohol or other substance use was channeled through unstructured socializing. Note that, unlike the coefficients in the previous tables, those in Table 6 are not transformed.

The key findings of this analysis are that the effects of the two single-parent families on heavy use were almost entirely indirect through unstructured socializing. Recall, for instance, that the odds ratio associated with single-mother families in Table 2 was 1.60. This can be considered the total effect without adjustment for other variables. The direct effect in Table 6 is only 0.04, which translates into an odds ratio of 1.04. However, the indirect effect due to unstructured socializing was larger, and its 95% CI did not include zero (coefficient = 0.10; 95% CI: 0.08, 0.12). A similar result occurred among single-father families and extended to heavy substance use as well.

Three direct effects remained though: even after accounting for unstructured socializing, youth from joint custody families were at elevated risk of heavy alcohol use; youth from mother–stepfather families were at elevated risk of heavy alcohol use or heavy substance use; and youth from families in which neither parent resided were at elevated risk of heavy substance use. Further, the association among youth who did not live with either parent and heavy alcohol use showed no mediation via unstructured socializing.

The results of the bootstrap method displayed in Table 6 were largely confirmed by the KHB method [70] (results not shown). For example, the mediating effect of unstructured socializing accounted for more than two-thirds of the effect of single-mother families and all the effect of single-father families on heavy alcohol use and on heavy substance use.

In general, the results suggested most plainly that unstructured socializing mediates almost completely the association between living in single-parent families and a heightened risk of heavy alcohol and substance use. On the other hand, youth living in mother–stepfather or joint custody families demonstrated an elevated risk of heavy alcohol or substance use directly and indirectly via unstructured socializing. Yet, youth who lived with neither parent were at higher risk of heavy substance use independent of unstructured socializing.

It might seem curious that the source of the reduction of effects among neither-parent families was not unstructured socializing. Though not within the purview of this research, a supplementary multilevel SEM suggested peer substance use might mediate a substantial portion of the association: youth from families in which neither parent resides may have been at higher risk, in general, of heavy substance use because they were likely to have friends who were substance users.

## 4. Discussion

Research conducted over the last 25 years has demonstrated a consistent association between family structure and adolescent substance use, including heavier and disordered forms of use [19,22,23,24,25]. Though the effect sizes are modest, studies using data from across the globe have shown that youth who live with a single parent, a stepparent, or no parents are at heightened risk of substance use, including alcohol, cannabis, and other substances. Since the associations are well-established, though, why should research continue to explore this topic? For one, the number of youth living in families without a mother or father has increased throughout much of the world [28,29]. Second, problem substance use among adolescents has also shown an uptick in recent years [2]. Third, most experts do not believe that family structure leads directly or uniformly to substance use; rather, studies have continued to search for factors that mediate or otherwise account for the effects of family living arrangements. The most frequently studied factors include economic resources, parent–child relationships, parental supervision, and stressful experiences [24,30]. Yet, none of these has sufficiently explained the link between family structure and adolescent substance use [22].

Building on recent research that suggests alternative pathways from family structure to substance use [22] and yet focusing on heavy forms of alcohol and substance use since they have been shown to affect life course problems [7,51], this study’s objective was to determine whether unstructured socializing mediated the association between family structure and heavy substance use. Unstructured socializing refers to time spent with friends outside the presence of adults but with no specific plans or structure [43,44,45]. Although this concept is related to both parental supervision and peer substance use, which are two consistent predictors of youth substance use [41,46], unstructured socializing is based on different assumptions regarding youth leisure time and agency. First, it recognizes that the context within which peer relations take place is important. Second, it posits that although parental supervision tends to be outside the direct control of youth, the way they spend their time is more flexible and agentic. Certainly, parents can exert control over the way their adolescent children spend time outside the home, but most youth are allowed some leeway to manage their time. Those who spend time in unstructured activities, especially when they associate with other youth, are at risk of behaviors such as heavy alcohol and substance use [47,48,73], and given that single parents and stepparents often do not have as many economic and social resources as two biological parents to invest in youth [33,34,35], including keeping track of their activities outside the home, it is reasonable to argue that unstructured socializing mediates the association between family structure and substance use.

Using a large dataset with information from youth residing in 30 nations in Europe and the Americas, the results of a series of empirical models supported the hypothesis that family structure effects on heavy alcohol and substance use are partly channeled through unstructured socializing. Among single-mother and single-father families, the mediation effect was almost complete. The findings also suggested a partial mediation effect of unstructured socializing for youth residing in mother–stepfather and joint custody families, with about one-quarter of the former’s association with heavy alcohol or substance use but only about one-tenth of the latter’s association with heavy alcohol use accounted for.

An exception occurred among neither-parent families. The higher risk of heavy substance use—which included use of cannabis, ecstasy, LDS, heroin, or similar psychoactives—among youth living with neither parent persisted after adjusting for the effects of unstructured socializing. Again, the risk was diminished with statistical adjustment for other variables, but unstructured socializing did not serve as a mediator among these youth. A complication is that adolescents who did not reside with either parent could have lived in numerous other arrangements, including with relatives or in foster families. Thus, understanding why these youth were at high risk of heavy substance use remains elusive.

In general, the results of the analyses supported the hypothesis with respect to single parents and point toward an intervention strategy that could reduce heavier or problematic forms of adolescent substance use. That is, the effectiveness of school and community programs designed to assist single parents could be enhanced by emphasizing youth activities outside the home. Many programs include activities for youth, but most public resources go toward providing single parents with financial support, job training and access, housing, and childcare [74]. Though these are undoubtedly important, too few resources are targeted at adolescents residing with single parents. Intervention efforts should be aimed at helping young people avoid problems with illicit substances and other deviant behaviors by furnishing positive activities at school and in the community as well as discouraging youth from merely hanging out with little purpose and less supervision.

### Study Limitations and Future Directions

Although the results offered compelling evidence that unstructured socializing mediates the association between living with a single parent and heavy alcohol or substance use, the research had several limitations. First, the data were cross-sectional, so the temporal order of the variables could not be identified. For example, heavy use and unstructured socializing might be associated in a reciprocal manner. It seems likely that youth who wish to use illicit substances look for opportunities to be with like-minded friends in unsupervised settings, and as use continues, seeking these environments probably continues.

Second, the data were collected about 15 years ago and may not reflect more recent patterns of substance use, including the decline of youth alcohol use and the increase in marijuana use across many nations [2,11,12,13]. The measures of heavy alcohol and other substance use, though used in previous studies that relied on the ISRD-2 data [55], were not based on scales that have been validated in previous research [56]. Thus, while the questions employed to instantiate substance use were similar to those used in other large national and international surveys, their measurement properties could not be fully ascertained.

Third, the measure of family structure was limited and may be affected by classification bias since only the youth were asked about current living arrangements. Information was also not available regarding when a particular family arrangement materialized. The ISRD-2 question asked only “Are you living with your own mother and father” and then offered various options to respondents about their living situations. However, respondents were not asked, for instance, when they became part of a single parent or stepparent family. Again, the temporal ordering of the variables in the empirical models was uncertain: heavy alcohol use or experiences with unstructured socializing may have preceded changes in family structure. Longitudinal data are necessary to sort through these issues [32]. Furthermore, the measure of family structure in the ISRD-2 could not be used to identify extended or alternative family structures, such as single parent + grandparent families, cohabiting relationships, or same-sex unions. Yet, having grandparents and other extended family members residing in the home may benefit adolescent health and development [75]. In general, then, the measure of family structure was limited.

Fourth, the ISRD-2 data were collected in two stages, but only one of these was random. First-stage collection involved a nonrandom selection of towns and cities in the nations, followed by a random selection of students from schools in these towns and cities. Therefore, although the sample size was impressive, it could not compensate for the lack of random selection during the first stage. This militates against inferential claims from the results to the nations’ young people in toto. Nonetheless, the consistency of the general results with those from studies of family structure and substance use that used different multination datasets [19,22,24] increases confidence that the findings offer a reasonable picture of youth substance use.

Additional research is needed to validate the findings of the current study, however. Longitudinal data with a more nuanced measure of family structure that features additional living arrangements such as extended kinship networks (e.g., grandparents) would help illuminate the processes by which family structure affects substance use and its proximal predictors. Moreover, attention to how family functioning differs across disparate family structures and what implications this has for adolescent substance use continues to be relevant. The present study simply adjusted empirically for the effects of some family functioning variables but did not explicitly identify their variation by family structure.

Data from non-Western nations, such as those in the Global South, would also be useful for exploring how family structure influences youth development cross-culturally, including its association with substance use [76]. Finally, studies are needed that address gender and ethnic differences. Heavy substance use is more common among adolescent boys, and girls’ use is often influenced by romantic partners [77,78], which could be an important consideration when studying the effects of unstructured socializing. Research has also identified ethnic differences in the prevalence of certain family structures and the risks of substance use [79,80]. This area of study could be gainfully expanded by considering mediating factors such as family relationships, peer networks, and unstructured socializing.

## 5. Conclusions

Numerous studies have shown that adolescents living with single parents, stepparents, or with neither parent are at risk of substance use, including heavier and more consequential forms of use. Yet, research has not clearly identified the factors that link family structure to substance use. Most studies have addressed parent–child relationships or stressful life events as confounders or mediators of this association. The present research demonstrated that unstructured socializing, a concept found in studies of juvenile delinquency, mediated almost all the association between living with a single parent and heavy alcohol or heavy substance use. If confirmed by other research, this result might be useful in developing more effective programs designed to avert detrimental forms of substance use by youth.

## Figures and Tables

**Table 1:** Descriptive statistics, adolescents ages 12–18, ISRD-2 variables, 2005–2007.

Variable	Percent or Mean	Std. Err.	Min–Max	Lower 95% CI	Upper 95% CI
Alcohol use categories					
Never used	36.50%	2.8		30.1	42.5
Used more than a month ago	48.1	2.3		43.3	52.9
Used in past month, not heavy	5.6	0.5		4.6	6.7
Heavy alcohol use in the past month	9.9	1		8.1	12
Substance use categories					
Never used	90.80%	1.1		88.3	92.8
Used more than a month ago	4.9	0.7		3.7	6.5
Used in past month, not heavy	1.8	0.3		1.3	2.5
Heavy use in the past month	2.5	0.4		1.7	3.6
Family type					
Mother–father	72.20%	1.7		68.5	75.6
Joint custody	5.1	0.9		3.6	7.1
Single mother	11.6	0.8		10.1	13.4
Single father	1.3	0.1		1.1	1.6
Mother–stepfather	6.1	0.6		5.1	7.4
Father–stepmother	0.8	0.1		0.7	1
Neither parent	0.7	0.1		0.5	1
Other family type	2.2	0.3		1.6	2.9
Male	48.00%	0.03		47.2	48.7
Age	13.43	0.08	12–18	13.27	13.59
Immigrant	7.70%	1.17		5.4	10.1
Parent is immigrant	20.50%	2.67		15.1	26
Neighborhood quality	76.71	0.7	0–100	75.28	78.09
Low economic resources	0.62	0.08	0–4	0.45	0.78
Parent–child relationship ^a^	0	0.05	−3.74–1.75	−0.13	0.12
Family involvement ^a^	0	0.05	−2.76–1.28	−0.55	0.73
Parental supervision ^a^	0	0.03	−2.57–1.62	−0.05	0.04
Stressful life events	1.56	0.04	0–9	1.47	1.64
Parent drug problems	7.60%	0.63		6.3	9
Low self-control ^a^	0	0.08	−3.87–4.99	−0.18	0.12
Peer substance use (logged)	0.38	0.04	0–2.56	0.3	0.46
Unstructured socializing ^a^	0	0.06	−2.44–2.67	−0.13	0.12
Country-level variables					
Percent single parent	18.10%	1.12	7.9–29.7	15.8	20.4
Liters of alcohol per capita	8.96	0.59	3.73–15.52	7.74	10.17
Percent cannabis users	5.40%	0.57	1.7–13.0	4.3	6.6

Note: The statistics are based on weighted data and adjusted for the complex sampling design of the ISRD-2. The sample size is 65,737. ISRD-2, Second International Self-Report Study; Std. err., standard error; CI, confidence interval. ^a^ Latent variable based on a polychoric principal components analysis (PCA) designed for categorical observed variables [58].

**Table 2:** Multilevel SEM of heavy alcohol use among adolescents, ISRD-2, 2005–2007.

Explanatory Variable	Model 1		Model 2	
	Odds Ratio	95% CI	Odds Ratio	95% CI
Family type ^a^				
Joint custody	1.81 *	1.33, 2.48	1.45 *	1.04, 2.02
Single mother	1.60 *	1.38, 1.86	1.04	0.84, 1.29
Single father	1.83 *	1.32, 2.53	0.92	0.67, 1.27
Mother–stepfather	2.46 *	1.92, 3.14	1.60 *	1.30, 1.97
Father–stepmother	1.74 *	1.19, 2.55	1.23	0.82, 1.87
Neither parent	2.42 *	1.52, 3.85	1.16	0.78, 1.73
Other family type	1.45 *	1.07, 1.97	0.96	0.64, 1.39
Male			1.07	0.94, 1.22
Age			1.25 *	1.16, 1.34
Immigrant			0.82	0.64, 1.04
Parent is immigrant			0.59 *	0.39, 0.88
Neighborhood quality			0.99	0.98, 1.01
Low economic resources			0.72 *	0.59, 0.87
Parent–child relationship			0.94	0.81, 1.07
Family involvement			0.69 *	0.60, 0.78
Parental supervision			0.76 *	0.67, 0.86
Stressful life events			1.05	0.98, 1.10
Parent drug problems			1.22 *	1.01, 1.49
Low self-control			1.30 *	1.22, 1.38
Peer substance use (logged)			4.51 *	4.06, 5.01
Unstructured socializing			2.06 *	1.84, 2.32
Country-level variables				
Percent single parent			0.99	0.94, 1.05
Liters of alcohol per capita			1.05	0.95, 1.15
Percent cannabis users			0.92	0.80, 1.05
Country-level variance	0.52	0.31, 0.86	0.43	0.26, 0.71
True-positive rate (TPR)	0.33		0.78	

Note: The results are based on a multinomial logistic SEM with no alcohol use as the reference category. Alcohol use was measured as a multinomial variable. See Table 1 for the categories of this variable. The results in this table are for the category heavy alcohol use in the past month only. Results for the other categories are available upon request. The odds ratios and 95% CIs are based on weighted data and adjusted for the complex sampling design of the ISRD-2. The sample size is 65,737. SEM, structural equation model; CI, confidence interval. ^a^ Mother–father families serve as the reference category. * 95% CI does not include zero.

**Table 3:** Multilevel SEM of heavy substance use among adolescents, ISRD-2, 2005–2007.

Explanatory Variable	Model 1		Model 2	
	Odds Ratio	95% CI	Odds Ratio	95% CI
Family type ^a^				
Joint custody	1.62 *	1.05, 2.50	1.30	0.93, 1.84
Single mother	1.73 *	1.30, 2.30	1.10	0.90, 1.35
Single father	2.45 *	1.68, 3.58	1.21	0.81, 1.80
Mother–stepfather	1.84 *	1.33, 2.55	1.38 *	1.11, 1.72
Father–stepmother	1.61	0.97, 2.65	1.26	0.73, 2.20
Neither parent	4.13 *	2.40, 7.11	2.45 *	1.43, 4.20
Other family type	1.39	0.78, 2.46	0.87	0.56, 1.41
Male			2.09 *	1.73, 2.52
Age			1.18 *	1.10, 1.26
Immigrant			0.85	0.65, 1.12
Parent is immigrant			1.09	0.88, 1.35
Neighborhood quality			0.98 *	0.97, 0.99
Low economic resources			1.12	0.99, 1.27
Parent-child relations			0.89 *	0.83, 0.95
Family involvement			0.96	0.86, 1.07
Parental supervision			0.82 *	0.76, 0.88
Stressful life events			1.01	0.93, 1.09
Parent drug problems			1.55 *	1.29, 1.85
Low self-control			1.28 *	1.20, 1.36
Peer substance use (logged)			4.51 *	3.72, 5.47
Unstructured socializing			1.62 *	1.37, 1.90
Country-level variables				
Percent single parent			0.99	0.95, 1.03
Liters of alcohol per capita			1.09	0.99, 1.20
Percent cannabis users			1.16 *	1.07, 1.25
Country-level variance	0.70	0.42, 1.17	0.21	0.12, 0.37
True-positive rate (TPR)	0.34		0.87	

Note: The results are based on a multinomial logistic SEM with no substance use as the reference category. Substance use was measured as a multinomial variable. See Table 1 for the categories of this variable. The results in this table are for the category heavy substance use in the past month only. Other results are available upon request. The odds ratios and 95% CIs are based on weighted data and adjusted for the complex sampling design of the ISRD-2. The sample size is 65,737. SEM, structural equation model; CI, confidence interval. ^a^ Mother–father families serve as the reference category. * 95% CI does not include zero.

**Table 4:** Multilevel SEM of unstructured socializing and heavy alcohol use among adolescents, ISRD-2, 2005–2007.

Explanatory Variable	Unstructured Socializing		Heavy Alcohol Use	
	LR Beta Weight ^a^	95% CI	Odds Ratio	95% CI
Family type ^b^				
Joint custody	0.09 *	0.01, 0.17	1.45 *	1.04, 2.02
Single mother	0.11 *	0.06, 0.15	1.04	0.84, 1.29
Single father	0.10 *	0.04, 0.15	0.92	0.67, 1.27
Mother–stepfather	0.16 *	0.10, 0.21	1.60 *	1.30, 1.97
Father–stepmother	−0.04	−0.13, 0.06	1.23	0.82, 1.87
Neither parent	−0.03	−0.18, 0.11	1.16	0.78, 1.73
Other family type	−0.01	−0.08, 0.07	0.96	0.64, 1.39
Male	−0.05	−0.11, 0.02	1.07	0.94, 1.22
Age	0.08 *	0.03, 0.12	1.25 *	1.16, 1.34
Immigrant	−0.05	−0.12, 0.01	0.82	0.64, 1.04
Parent is immigrant	−0.05	−0.14, 0.04	0.59 *	0.39, 0.88
Neighborhood quality	−0.01	−0.03, 0.01	0.99	0.98, 1.01
Parental supervision	−0.29 *	−0.32, −0.27	0.72 *	0.59, 0.87
Low economic resources			0.94	0.81, 1.07
Parent–child relations			0.69 *	0.60, 0.78
Family involvement			0.76 *	0.67, 0.86
Stressful life events			1.05	0.98, 1.10
Parent drug problems			1.22 *	1.01, 1.49
Low self-control			1.30 *	1.23, 1.39
Peer substance use (logged)			4.51 *	4.06, 5.01
Unstructured socializing			2.06 *	1.83, 2.32
Country-level variables				
Percent single parent	0.02	−0.05, 0.10	0.99	0.94, 1.05
Liters of alcohol per capita	0.06	−0.03, 0.15	1.05	0.95, 1.15
Percent cannabis users	−0.03	−0.09, 0.14	0.92	0.80, 1.05
Country-level variance	0.90		95% CI: 0.85, 0.96	

Note: The results are based on an SEM that simultaneously modeled unstructured socializing with a linear model and heavy alcohol use with a multinomial logistic model. In the latter model, no alcohol use was the reference category, but the results are shown for the category heavy alcohol use in the past month only. See Table 1 for the categories of this variable. Results for the other categories are available upon request. The coefficients and 95% CIs are based on weighted data and adjusted for the complex sampling design of the ISRD-2. The sample size is 65,737. LR, linear regression; SEM, structural equation model; CI, confidence interval. ^a^ Standardized coefficients are presented. Fully standardized coefficients are shown when the explanatory variable is continuous, and *y*-standardization coefficients are shown when the explanatory variable is categorical. ^b^ Mother–father families serve as the reference category. * 95% CI does not include zero.

**Table 5:** Multilevel SEM of unstructured socializing and heavy substance use among adolescents, ISRD-2, 2005–2007.

Explanatory Variable	Unstructured Socializing		Heavy Substance Use	
	LR Beta Weight ^a^	95% CI	Odds Ratio	95% CI
Family type ^b^				
Joint custody	0.09 *	0.01, 0.17	1.29	0.93, 1.84
Single mother	0.11 *	0.06, 0.15	1.10	0.90, 1.35
Single father	0.10 *	0.04, 0.15	1.21	0.81, 1.80
Mother–stepfather	0.16 *	0.10, 0.21	1.39 *	1.11, 1.72
Father–stepmother	−0.04	−0.13, 0.06	1.26	0.73, 2.20
Neither parent	−0.03	−0.18, 0.11	2.45 *	1.43, 4.20
Other family type	−0.01	−0.08, 0.07	0.87	0.56, 1.41
Male	−0.05	−0.11, 0.02	2.10 *	1.74, 2.53
Age	0.08 *	0.03, 0.12	1.18 *	1.10, 1.26
Immigrant	−0.05	−0.12, 0.01	0.85	0.65, 1.12
Parent is immigrant	−0.05	−0.14, 0.04	1.09	0.88, 1.35
Neighborhood quality	−0.01	−0.03, 0.01	0.98 *	0.97, 0.99
Parental supervision	−0.29 *	−0.32, −0.27	1.12	0.99, 1.27
Low economic resources			0.89 *	0.83, 0.95
Parent–child relationship			0.96	0.86, 1.07
Family involvement			0.82 *	0.76, 0.88
Stressful life events			1.01	0.93, 1.09
Parent drug problems			1.55 *	1.29, 1.85
Low self-control			1.28 *	1.20, 1.36
Peer substance use (logged)			4.51 *	3.72, 5.47
Unstructured socializing			1.62 *	1.37, 1.90
Country-level variables				
Percent single parent	0.02	−0.05, 0.10	0.99	0.95, 1.03
Liters of alcohol per capita	0.06	−0.03, 0.15	1.09	0.99, 1.20
Percent cannabis users	−0.03	−0.09, 0.14	1.16 *	1.07, 1.25
Country-level variance	0.91		95% CI: 0.86, 0.95	

Note: The results are based on an SEM that simultaneously modeled unstructured socializing with a linear model and heavy substance use with a multinomial logistic model. In the latter model, no substance use was the reference category, but the results are shown for the category heavy substance use in the past month only. See Table 1 for the categories of this variable. Results for the other categories are available upon request. The coefficients and 95% CIs are based on weighted data and adjusted for the complex sampling design of the ISRD-2. The sample size is 65,737. LR, linear regression; SEM, structural equation model; CI, confidence interval. ^a^ Standardized coefficients are presented. Fully standardized coefficients are shown when the explanatory variable is continuous, and *y*-standardization coefficients are shown when the explanatory variable is categorical. ^b^ Mother–father families serve as the reference category. * 95% CI does not include zero.

**Table 6:** Direct and indirect effects via unstructured socializing of family structure on heavy alcohol use and heavy substance use among adolescents, ISRD-2, 2005–2007.

	Unstructured Socializing		Heavy Alcohol Use			
			Direct Effect		Indirect Effect	
	Coefficient	95% CI	Coefficient	95% CI	Coefficient	95% CI
Family type ^a^						
Joint custody	0.75 *	−0.03, 0.18	0.37 *	0.22, 0.52	0.05 *	0.02, 0.08
Single mother	0.14 *	0.09, 0.20	0.04	−0.10, 0.18	0.10 *	0.08, 0.12
Single father	0.12 *	0.05, 0.20	−0.08	−0.39, 0.23	0.07 *	0.02, 0.16
Mother–stepfather	0.20 *	0.11, 0.28	0.48 *	0.32, 0.63	0.14 *	0.11, 0.17
Father–stepmother	−0.05	−0.16, 0.05	0.21	−0.20, 0.62	−0.03	−0.12, 0.05
Neither parent	−0.08	−0.28, 0.14	0.18	−0.29, 0.64	−0.05	−0.13, 0.02
Other family type	−0.01	−0.14, 0.14	−0.06	−0.35, 0.25	−0.01	−0.35, 0.23
Unstructured socializing			0.71 *	0.60, 0.83		
	**Unstructured Socializing**		**Heavy Substance Use**			
			**Direct Effect**		**Indirect Effect**	
	**Coefficient**	**95% CI**	**Coefficient**	**95% CI**	**Coefficient**	**95% CI**
Family type ^a^						
Joint custody	0.08	−0.02, 0.18	0.27	−0.07, 0.62	0.03 *	0.01, 0.06
Single mother	0.14 *	0.09, 0.20	0.10	−0.07, 0.27	0.07 *	0.05, 0.08
Single father	0.12 *	0.05, 0.20	0.19	−0.19, 0.57	0.10 *	0.04, 0.15
Mother–stepfather	0.20 *	0.11, 0.28	0.32 *	0.07, 0.57	0.09 *	0.07, 0.11
Father–stepmother	−0.05	−0.16, 0.05	0.23	−0.31, 0.78	−0.02	−0.08, 0.03
Neither parent	−0.08	−0.29, 0.14	0.90 *	0.41, 1.40	−0.03	−0.08, 0.02
Other family type	−0.01	−0.14, 0.14	−0.12	−0.59, 0.25	−0.01	−0.03, 0.03
Unstructured socializing			0.46 *	0.29, 0.64		

Note: The coefficients are untransformed estimates from an SEM that utilized a linear model to predict unstructured socializing and a multinomial logistic model to simultaneously predict alcohol use or illicit substance use since the latter two were measured as multinomial variables. The SEM included statistical adjustments for the other variables listed in Table 2, Table 3, Table 4 and Table 5. The results shown in the table are for the categories of heavy alcohol use and heavy substance use in the past month only (see Table 1 for a list of the other categories). Results for the other alcohol or substance use categories are available upon request. The coefficients and 95% CIs were estimated via the bootstrap (250 iterations), with the percentile method utilized [71], and were adjusted for the complex sampling design of the ISRD-2. The sample size is 65,737. SEM, structural equation model; CI, confidence interval. ^a^ Mother–father families serve as the reference category. * 95% CI does not include zero.

## Data Availability

The data were accessed by the author from the Inter-university Consortium for Political and Social Research (www.icpsr.umich.edu (accessed on 12 June 2022)). The author was not involved in the data collection, management, or administration.

## Appendix A

**Table A1:** Percentage of adolescents, ages 12–18, reporting heavy alcohol use and heavy substance use, by country, ISRD-2, 2005–2007.

Country	Heavy Alcohol Use (%)	Heavy Substance Use (%)	Sample Size
Armenia	2.51	0.10	2005
Aruba	8.50	1.13	600
Austria	14.01	1.17	2738
Belgium	10.40	8.41	192
Bosnia and Herzegovina	2.90	0.45	1779
Cyprus	5.60	2.38	2022
Czech Republic	9.41	2.53	3027
Denmark	20.15	1.96	1171
Estonia	16.31	1.99	2327
Finland	18.05	0.07	1342
France	3.92	9.50	2663
Germany	15.94	1.55	3090
Hungary	7.83	1.54	2073
Iceland	4.01	0.34	533
Ireland	17.00	4.29	1434
Italy	7.73	3.18	6570
Lithuania	10.40	1.52	1936
The Netherlands	14.78	2.58	2109
NL Antilles	5.23	2.15	1532
Norway	10.92	1.06	1458
Poland	14.12	2.03	1925
Portugal	4.30	0.54	2488
Russia	5.25	6.18	2210
Slovenia	7.89	1.43	2021
Spain	16.56	10.23	3478
Suriname	4.32	1.17	2120
Sweden	8.30	0.88	1992
Switzerland	12.45	3.76	3289
United States	4.67	5.13	2116
Venezuela	7.61	1.72	1767
Total	9.91	2.54	65,737

Note: The percentages are based on weighted data and adjusted for the complex sampling design of the ISRD-2.

## References

[1] De la Torre-Luque A., Ozeylem F., Essau C.A. (2021). Prevalence of addictive behaviours among adolescents from 73 low-and middle-income countries. Addict. Behav. Rep..

[2] United Nations Office on Drugs and Crime (UNODC) (2018). Drugs and Age: Drugs and Associated Issues Among Young People and Older People. https://www.unodc.org/wdr2018/prelaunch/WDR18_Booklet_4_YOUTH.pdf.

[3] Albaugh M.D., Ottino-Gonzalez J., Sidwell A., Lepage C., Juliano A., Owens M.M., Chaarani B., Spechler P., Fontaine N., Rioux P. (2021). Association of cannabis use during adolescence with neurodevelopment. JAMA Psychiatry.

[4] Davis J.P., Dumas T.M., Merrin G.J., Espelage D.L., Tan K., Madden D., Hong J.S. (2018). Examining the pathways between bully victimization, depression, academic achievement, and problematic drinking in adolescence. Psychol. Addict. Behav..

[5] Gubbels J., van der Put C.E., Assink M. (2019). Risk factors for school absenteeism and dropout: A meta-analytic review. J. Youth Adolesc..

[6] Marshall E.J. (2014). Adolescent alcohol use: Risks and consequences. Alcohol Alcohol..

[7] Squeglia L.M., Tapert S.F., Sullivan E.V., Jacobus J., Meloy M.J., Rohlfing T., Pfefferbaum A. (2015). Brain development in heavy-drinking adolescents. Am. J. Psychiatry.

[8] Thompson K., Holley M., Sturgess C., Leadbeater B. (2021). Co-use of alcohol and cannabis: Longitudinal associations with mental health outcomes in young adulthood. Int. J. Environ. Res. Public Health.

[9] American Academy of Child & Adolescent Psychiatry (2019). Marijuana and Teens. https://www.aacap.org/AACAP/Families_and_Youth/Facts_for_Families/FFF-Guide/Marijuana-and-Teens-106.aspx.

[10] World Health Organization (WHO) (2016). The Health and Social Effects of Nonmedical Cannabis Use. http://apps.who.int/iris/rest/bitstreams/1065247/retrieve.

[11] Vos T., Lim S.S., Abbafati C., Abbas K.M., Abbasi M., Abbasifard M., Abbasi-Kangevari M., Abbastabar H., Abd-Allah F., Abdelalim A. (2020). Global burden of 369 diseases and injuries in 204 countries and territories, 1990–2019: A systematic analysis for the Global Burden of Disease Study 2019. Lancet.

[12] Leal-López E., Sánchez-Queija I., Vieno A., Currie D., Torsheim T., Pavlova D., Moreno-Maldonado C., De Clercq B., Kalman M., Inchley J. (2021). Cross-national time trends in adolescent alcohol use from 2002 to 2014. Eur. J. Public Health.

[13] Vashishtha R., Livingston M., Pennay A., Dietze P., MacLean S., Holmes J., Herring R., Caluzzi G., Lubman D.I. (2020). Why is adolescent drinking declining? A systematic review and narrative synthesis. Addict. Res. Theory.

[14] Allen J.P., Loeb E.L., Narr R.K., Costello M.A. (2021). Different factors predict adolescent substance use versus adult substance abuse: Lessons from a social-developmental approach. Dev. Psychopathol..

[15] Brumback T., Thompson W., Cummins K., Brown S., Tapert S. (2021). Psychosocial predictors of substance use in adolescents and young adults: Longitudinal risk and protective factors. Addict. Behav..

[16] Becoña E., Martínez Ú., Calafat A., Juan M., Duch M., Fernández-Hermida J.R. (2012). How does family disorganization influence children’s drug use? A review. Adicciones.

[17] Jokinen T., Alexander E.C., Manikam L., Huq T., Patil P., Benjumea D., Das I., Davidson L.L. (2021). A systematic review of household and family alcohol use and adolescent behavioural outcomes in low-and middle-income countries. Child Psychiatry Hum. Dev..

[18] Rusby J.C., Light J.M., Crowley R., Westling E. (2018). Influence of parent–youth relationship, parental monitoring, and parent substance use on adolescent substance use onset. J. Family Psychol..

[19] Bjarnason T., Andersson B., Choquet M., Elekes Z., Morgan M., Rapinett G. (2003). Alcohol culture, family structure and adolescent alcohol use: Multilevel modeling of frequency of heavy drinking among 15–16-year-old students in 11 European countries. J. Stud. Alcohol.

[20] Coles R.L. (2015). Single-father families: A review of the literature. J. Fam. Theory Rev..

[21] Ewing B.A., Osilla K.C., Pedersen E.R., Hunter S.B., Miles J.N., D’Amico E.J. (2015). Longitudinal family effects on substance use among an at-risk adolescent sample. Addict. Behav..

[22] Hoffmann J.P. (2017). Family structure and adolescent substance use: An international perspective. Subst. Use Misuse.

[23] Hoffmann J.P., Johnson R.A. (1998). A national portrait of family structure and adolescent drug use. J. Marriage Fam..

[24] Ledoux S., Miller P., Choquet M., Plant M. (2002). Family structure, parent–child relationships, and alcohol and other drug use among teenagers in France and the United Kingdom. Alcohol Alcohol..

[25] Zhang S., Lim Y., Boyas J.F., Burlaka V. (2020). Family structure and youth illicit drug use, use disorder, and treatment services utilization. Child. Youth Serv. Rev..

[26] Conway K.P., Vullo G.C., Nichter B., Wang J., Compton W.M., Iannotti R.J., Simons-Morton B. (2013). Prevalence and patterns of polysubstance use in a nationally representative sample of 10th graders in the United States. J. Adolesc. Health.

[27] Rüütel E., Sisask M., Värnik A., Värnik P., Carli V., Wasserman C., Hoven C.W., Sarchiapone M., Apter A., Balazs J. (2014). Alcohol consumption patterns among adolescents are related to family structure and exposure to drunkenness within the family: Results from the SEYLE project. Intern. J. Environ. Res. Pub. Health.

[28] United Nations Population Division (UNDP) (2019). Household Size and Composition.

[29] Heine S., The Rise of Single Motherhood in the EU: Analysis and Propositions (2016). European Policy Brief No. 42. https://www.egmontinstitute.be/content/uploads/2016/03/EPB42.pdf?type=pdf.

[30] Broman C.L., Li X., Reckase M. (2008). Family structure and mediators of adolescent drug use. J. Fam. Issues.

[31] Jarvis J.A., Gibby A.L., Dufur M.J., Pribesh S. (2020). Family structure and child well-being in a non-western context: The role of parent–child relations and parental conflict in South Korea. Popul. Res. Policy Rev..

[32] Jarvis J.A., Otero C., Poff J.M., Dufur M.J., Pribesh S.L. (2021). Family structure and child behavior in the United Kingdom. J. Child Fam. Stud..

[33] Thomson E., Hanson T.L., McLanahan S.S. (1994). Family structure and child well-being: Economic resources vs. parental behaviors. Soc. Forces.

[34] Painter M., Frech A., Williams K. (2015). Nonmarital fertility, union history, and women’s wealth. Demography.

[35] Ganong L.H., Coleman M. (2004). Stepfamily Relationships: Development, Dynamics, and Interventions.

[36] Van Eeden-Moorefield B., Pasley B.K., Peterson G.W., Bush K.R. (2013). Remarriage and stepfamily life. Handbook of Marriage and the Family.

[37] Lerner R.M., Castellino D.R. (2013). Adolescents and Their Families: Structure, Function, and Parent-Youth Relations.

[38] Hadfield K., Amos M., Ungar M., Gosselin J., Ganong L. (2018). Do changes to family structure affect child and family outcomes? A systematic review of the instability hypothesis. J. Fam. Theory Rev..

[39] Hoffmann J.P., Jones M.S. (2022). Cumulative stressors and adolescent substance use: A review of 21st-century literature. Trauma Violence Abus..

[40] Elkington K.S., Bauermeister J.A., Zimmerman M.A. (2010). Psychological distress, substance use, and HIV/STI risk behaviors among youth. J. Youth Adolesc..

[41] Henneberger A.K., Mushonga D.R., Preston A.M. (2021). Peer influence and adolescent substance use: A systematic review of dynamic social network research. Adolesc. Res. Rev..

[42] Felson M., Wortley L., Mazerolle L. (2016). The routine activity approach. Environmental Criminology and Crime Analysis.

[43] Osgood D.W., Anderson A.L. (2004). Unstructured socializing and rates of delinquency. Criminology.

[44] Hoeben E.M., Osgood D.W., Siennick S.E., Weerman F.M. (2021). Hanging out with the wrong crowd? The role of unstructured socializing in adolescents’ specialization in delinquency and substance use. J. Quant. Criminol..

[45] Hoeben E.M., Weerman F.M. (2016). Why is involvement in unstructured socializing related to adolescent delinquency?. Criminology.

[46] Beier H. (2018). Situational peer effects on adolescents’ alcohol consumption: The moderating role of supervision, activity structure, and personal moral rules. Deviant Behav..

[47] Hoeben E.M., Meldrum R.C., Walker D.A., Young J.T. (2016). The role of peer delinquency and unstructured socializing in explaining delinquency and substance use: A state-of-the-art review. J. Crim. Justice.

[48] Meldrum R.C., Leimberg A. (2018). Unstructured socializing with peers and risk of substance use: Where does the risk begin?. J. Drug Issues.

[49] Fisher P.A., Leve L.D., O’Leary C.C., Leve C. (2003). Parental monitoring of children’s behavior: Variation across stepmother, stepfather, and two-parent biological families. Fam. Relat..

[50] Tomčíková Z., Dankulincová Veselská Z., Madarasová Gecková A., van Dijk J.P., Reijneveld S.A. (2015). Adolescents’ drinking and drunkenness more likely in one-parent families and due to poor communication with mother. Cent. Eur. J. Public Health.

[51] Yuen W.S., Chan G., Bruno R., Clare P., Mattick R., Aiken A., Boland V., McBride N., McCambridge J., Slade T. (2020). Adolescent alcohol use trajectories: Risk factors and adult outcomes. Pediatrics.

[52] Junger-Tas J., Marshall I.H., Enzmann D., Killias M., Steketee M., Gruszczynska B. (2009). Juvenile Delinquency in Europe and Beyond: Results of the Second International Self-Report Delinquency Study.

[53] Junger-Tas J., Marshall I.H., Enzmann D., Killias M., Steketee M., Gruszczynska B. (2011). The Many Faces of Youth Crime.

[54] Hoffmann J.P., Bahr S.J. (2010). Estimating the prevalence and frequency of adolescent drug use: Do the models fit the measures?. J. Drug Issues.

[55] Innamorati M., Maniglio R. (2015). Psychosocial correlates of alcohol use and heavy episodic drinking among Italian adolescents: Data from the second international self-reported delinquency study. Am. J. Addict..

[56] Patrick M.E., Schulenberg J.E. (2010). Alcohol use and heavy episodic drinking prevalence and predictors among national samples of American eighth-and tenth-grade students. J. Stud. Alcohol Drugs.

[57] Felson R.B., Vanhee A.J. (2022). Situational peer effects on delinquency^1^. Justice Q..

[58] Kolenikov S., Angeles G. (2009). Socioeconomic status measurement with discrete proxy variables: Is principal component analysis a reliable answer?. Rev. Income Wealth.

[59] Flora D.B. (2020). Your coefficient alpha is probably wrong, but which coefficient omega is right? A tutorial on using R to obtain better reliability estimates. Adv. Methods Pract. Psychol. Sci..

[60] Kuder G.F., Richardson M.W. (1937). The theory of the estimation of test reliability. Psychometrika.

[61] Kuppens S., Moore S.C., Gross V., Lowthian E., Siddaway A.P. (2020). The enduring effects of parental alcohol, tobacco, and drug use on child well-being: A multilevel meta-analysis. Dev. Psychopathol..

[62] Vazsonyi A.T., Ksinan A.J., Javakhishvili M. (2021). Problems of cross-cultural criminology no more! Testing two central tenets of self-control theory across 28 nations. J. Crim. Justice.

[63] World Health Organization (WHO) (2007). Global Information System on Alcohol and Health. https://www.who.int/data/gho/data/themes/global-information-system-on-alcohol-and-health.

[64] United Nations Office on Drugs and Crime (UNODC) (2006). World Drug Report 2005. https://www.unodc.org/pdf/WDR_2005/volume_2_web.pdf.

[65] Hughes R.A., Heron J., Sterne J.A., Tilling K. (2019). Accounting for missing data in statistical analyses: Multiple imputation is not always the answer. Int. J. Epidemiol..

[66] Hox J.J., Little T.D. (2013). Multilevel regression and multilevel structural equation modeling. The Oxford Handbook of Quantitative Methods, Vol. 2.

[67] Janssen H.J., Weerman F.M., Eichelsheim V.I. (2017). Parenting as a protective factor against criminogenic settings? Interaction effects between three aspects of parenting and unstructured socializing in disordered areas. J. Res. Crime Delinq..

[68] Iacobucci D. (2012). Mediation analysis and categorical variables: The final frontier. J. Consum. Psychol..

[69] MacKinnon D.P., Lockwood C.M., Williams J. (2004). Confidence limits for the indirect effect: Distribution of the product and resampling methods. Multivar. Behav. Res..

[70] Breen R., Bernt Karlson K., Holm A. (2021). A note on a reformulation of the KHB method. Sociol. Methods Res..

[71] Falk C.F. (2018). Are robust standard errors the best approach for interval estimation with nonnormal data in structural equation modeling?. Struct. Equ. Modeling.

[72] Falk C.F., Biesanz J.C. (2015). Inference and interval estimation methods for indirect effects with latent variable models. Struct. Equ. Modeling.

[73] Leimberg A., Lehmann P.S. (2022). Unstructured socializing with peers, low self-control, and substance use. Int. J. Offender Ther. Comp. Criminol..

[74] European Commission (2019). Mechanisms Supporting Single Parents across the European Union. Directorate-General for Employment, Social Affairs and Inclusion, Geneva. https://op.europa.eu/en/publication-detail/-/publication/3ade5c22-b4d7-11e9-9d01-01aa75ed71a1/language-en.

[75] Attar-Schwartz S., Buchanan A. (2018). Grandparenting and adolescent well-being: Evidence from the UK and Israel. Contemp. Soc. Sci..

[76] Somefun O.D., Odimegwu C. (2018). The protective role of family structure for adolescent development in sub-Saharan Africa. PLoS ONE.

[77] Wilsnack R.W., Wilsnack S.C., Gmel G., Kantor L.W. (2018). Gender differences in binge drinking: Prevalence, predictors, and consequences. Alcohol. Res..

[78] Moon D.G., Hecht M.L., Jackson K.M., Spellers R.E. (1999). Ethnic and gender differences and similarities in adolescent drug use and refusals of drug offers. Subst. Use Misuse.

[79] Pew Research Center (2015). The American Family Today. https://www.pewresearch.org/social-trends/2015/12/17/1-the-american-family-today.

[80] Chen P., Jacobson K.C. (2012). Developmental trajectories of substance use from early adolescence to young adulthood: Gender and racial/ethnic differences. J. Adolesc. Health.

